# Predictive model for post-induction hypotension in patients undergoing transcatheter aortic valve implantation: a retrospective observational study

**DOI:** 10.1186/s40981-024-00717-0

**Published:** 2024-05-24

**Authors:** Kohei Noto, Satoshi Uchida, Hirotaka Kinoshita, Daiki Takekawa, Tetsuya Kushikata, Kazuyoshi Hirota

**Affiliations:** Department of Anesthesiology, Hirosaki University Graduate School of Medicine, Hirosaki, 036-8562 Japan

**Keywords:** Predict model, Post-induction hypotension, Transcatheter aortic valve implantation

## Abstract

**Purpose:**

Post-induction hypotension (PIH) is an independent risk factor for prolonged postoperative stay and hospital death. Patients undergoing transcatheter aortic valve implantation (TAVI) are prone to develop PIH. This study aimed to develop a predictive model for PIH in patients undergoing TAVI.

**Methods:**

This single-center retrospective observational study included 163 patients who underwent TAVI. PIH was defined as at least one measurement of systolic arterial pressure <90 mmHg or at least one incident of norepinephrine infusion at a rate >6 µg/min from anesthetic induction until 20 min post-induction. Multivariate logistic regression analysis was performed to develop a predictive model for PIH in patients undergoing TAVI.

**Results:**

In total, 161 patients were analyzed. The prevalence of PIH was 57.8%. Multivariable logistic regression analysis showed that baseline mean arterial pressure ≥90 mmHg [adjusted odds ratio (aOR): 0.413, 95% confidence interval (95% CI): 0.193–0.887; *p*=0.023] and higher doses of fentanyl (per 1-µg/kg increase, aOR: 0.619, 95% CI: 0.418–0.915; *p*=0.016) and ketamine (per 1-mg/kg increase, aOR: 0.163, 95% CI: 0.062–0.430; *p*=0.002) for induction were significantly associated with lower risk of PIH. A higher dose of propofol (per 1-mg/kg increase, aOR: 3.240, 95% CI: 1.320–7.920; *p*=0.010) for induction was significantly associated with higher risk of PIH. The area under the curve (AUC) for this model was 0.802.

**Conclusion:**

The present study developed predictive models for PIH in patients who underwent TAVI. This model may be helpful for anesthesiologists in preventing PIH in patients undergoing TAVI.

## Introduction

Aortic stenosis (AS), which can cause not only heart frailty but also sudden death, is the most common heart valve disease in developed countries with an aging population [1]. Although surgical aortic valve replacement (SAVR) has been the standard treatment for decades, transcatheter aortic valve implantation (TAVI) has become an alternative treatment for elderly patients with severe AS at moderate and high operative risk over the last 10 years [2, 3]. Thus, elderly patients with severe aortic stenosis with many comorbidities who are not candidates for SAVR can now be treated with TAVI. However, as it is difficult to maintain the hemodynamics of these patients after anesthetic induction and during the operation, anesthetic management of these patients is challenging for anesthesiologists.

The prevalence of post-induction hypotension (PIH) is reported to be 9.0–36.5% even though the definition of PIH and the patients included in each study differed [4–6]. As PIH is an independent risk factor for prolonged postoperative stay and hospital death [4, 7], it is important for anesthesiologists to predict and prevent the development of PIH. Previous studies have shown that lower baseline arterial pressure, older age, American Society of Anesthesiologists physical status (ASA-PS) III or IV, and the presence of diabetes mellitus type II were associated with a higher risk of development of PIH according to patients’ background [4–6]. Additionally, the use of propofol for anesthetic induction and increasing the induction dosage of fentanyl were also associated with a higher risk of developing PIH with regard to the anesthetic method [4]. However, as these studies were mostly patients with ASA-PS I or II and were younger than patients undergoing TAVI, these results may not be applicable to patients undergoing TAVI. Thus, developing predictive models for PIH in patients undergoing TAVI is needed, which would help anesthesiologists prevent the development of PIH and improve postoperative outcomes in patients undergoing TAVI.

This study aimed to develop a predictive model for PIH in patients undergoing TAVI. In addition, we evaluated the association between PIH and postoperative outcomes in patients undergoing TAVI.

## Methods

### Study procedures and patients

This single-center, retrospective observational study was approved by the Ethics Committee of the Hirosaki University Graduate School of Medicine, Hirosaki, Japan and was published on our department and hospital homepage (2022–063). The requirement for written informed consent from each patient was waived because of the retrospective nature of the study, and the Ethics Committee approved the waiver.

We included 163 patients who underwent TAVI at Hirosaki University Hospital between November 5, 2019 and August 9, 2022. Patients who underwent TAVI with monitored anesthesia care (MAC) were excluded.

PIH was defined as at least one measurement of systolic arterial pressure (SAP) <90 mmHg or at least one incident of norepinephrine infusion at a rate >6 µg/min from anesthetic induction until 20 min post-induction, which is same as a previous study [5].

Patients with PIH were assigned to the PIH group and those without PIH were assigned to the non-PIH group.

### Data collection

The following data were obtained from the medical and anesthesia records: age, sex, body mass index, ASA-PS, New York Heart Association (NYHA) functional class, Society of Thoracic Surgeons (STS) risk score, past medical history, type of antihypertensive agents, cardiac echocardiogram data, amount of anesthetics for induction, amount of vasopressor use, heart rate (HR) (pre-induction, minimum from induction of general anesthesia until 20 min post-induction), duration of anesthesia and surgery, amount of intraoperative blood loss, amount of intraoperative urine output, amount of intraoperative fluid infusion, length of intensive care unit (ICU) stay, and length of hospital stay.

### Anesthetic induction procedure

All patients were premedicated with 75 mg roxatidine acetate hydrochloride. All antihypertensive agents were continued on the day of the surgery. A radial arterial line was placed prior to anesthetic induction in all cases. The choice of anesthetic was not standardized and was performed by an anesthesiologist. For anesthetic induction, we used a combination of propofol or remimazolam, remifentanil, and/or fentanyl, with or without ketamine and rocuronium. For maintenance anesthesia, we used a combination of propofol or remimazolam, remifentanil, and/or fentanyl, with or without ketamine and rocuronium, or a combination of desflurane and remifentanil with/without fentanyl and rocuronium.

### Statistical analyses

The patients’ characteristic data, intraoperative data, and postoperative outcomes were presented as the median (25^th^ to 75^th^ percentile) and the number (percentage of each group). Statistical differences between the two groups were assessed using Fisher’s exact test for categorical variables and the Mann–Whitney U test for continuous variables.

We performed a multivariable logistic regression analysis to develop a predictive model for PIH in patients undergoing TAVI. To estimate the optimal cutoff value of continuous variables for predicting the development of PIH in multivariate logistic regression analyses, a receiver operating characteristic (ROC) curve analysis was conducted for each continuous variable. The STS risk score was adjusted for age, sex, comorbidities, and cardiac function. Baseline blood pressure, age, ASA-PS, and the presence of type II diabetes mellitus have been reported to be associated with a higher risk of developing PIH [4–6]. However, as age and the presence of type II diabetes mellitus were used to calculate STS risk scores, these variables were not included. Additionally, ASA-PS was also not included because 96.3% of patients in the study were ASA-PS 3. Therefore, only the baseline blood pressure was included. A mean pressure gradient of ≥60 mmHg is reported to be associated with an increased risk of severe AS [8] and was included in the definition of very severe AS [9]. Thus, as a mean pressure gradient of ≥60 mmHg may be associated with PIH, it was also included. The type and amount of anesthesia used for induction were also included. The variance inflation factor (VIF) was used to check for multicollinearity among the variables. Discrimination was measured using the area under the curve (AUC). The results are expressed as adjusted odds ratios (aORs) with corresponding 95% confidence intervals (CIs).

Additionally, we performed Kaplan–Meier curve analysis with the log-rank test to investigate the effect of PIH on the length of hospital stay, and we compared the probability of hospital stay between the PIH and non-PIH groups.

All data analyses were performed using EZR software ver. 1.61 (Saitama Medical Center, Jichi Medical University, Saitama, Japan). Statistical significance was set at *p* < 0.05.

## Results

**Fig 1:**
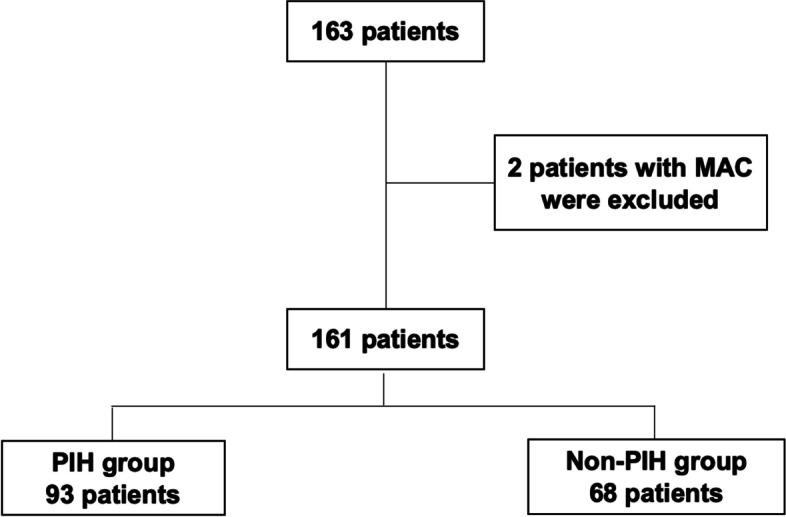
Flow chart of this study cohort. MAC: monitored anesthesia care, PIH: post-induction hypotension

Of 163 patients, 161 were finally analyzed after exclusion (Fig. 1), with 93 and 68 patients assigned to the PIH and non-PIH groups, respectively. The prevalence of PIH in patients who underwent TAVI was 57.8% in the present study. There are not any patients who needed norepinephrine infusion at a rate >6 µg/min from anesthetic induction until 20 min post-induction.

Patient characteristics are shown in Table 1. There were no significant differences between the two groups. Perioperative data of the patients are presented in Table 2. The doses of propofol [PIH group vs. non-PIH group, median (25^th^ to 75^th^ percentile); 0.44 (0.00, 0.88) mg/kg vs. 0.00 (0.00, 0.73) mg/kg, *p*=0.016] and remifentanil [0.20 (0.10, 0.20) µg/kg/min vs. 0.10 (0.00, 0.20) µg/kg/min, p=0.002] were higher in the PIH group than in the non-PIH group. The doses of ketamine [0.45 (0.00, 0.74) mg/kg vs. 0.79 (0.37, 1.00) mg/kg, *p*<0.001] and fentanyl [0.00 (0.00, 1.02) µg/kg vs. 1.07 (0.00, 1.74) µg/kg, *p*<0.001] were lower in the PIH group than in the non-PIH group. In the present study, the maximum doses of fentanyl, ketamine, and propofol were 4.27 µg/kg, 2.10 mg/kg, and 1.71 mg/mg, respectively. No significant differences were observed between the type of valve, duration of surgery and anesthesia, blood loss, urine output, fluid infusion, length of ICU and hospital stay, hospital death, and perioperative stroke. Hemodynamic parameters from induction of general anesthesia until 20 min post-induction are shown in Table 3. The baseline SAP, mean arterial pressure (MAP), and diastolic arterial pressure (DAP) were significantly lower in the PIH group than in the non-PIH group. The minimum SAP, MAP, and DAP before and after tracheal intubation were significantly lower in the PIH group than in the non-PIH group. The minimum HR after tracheal intubation was significantly lower in the PIH group than that in the non-PIH group. The amounts of ephedrine and phenylephrine used during this period were significantly higher in the PIH group than in the non-PIH group.

**Table 1:** Patients’ characteristics

	**PIH group**	**Non-PIH group**	***P* value**
N	93	68	N.A.
Male, n	35 (37.6%)	20 (29.4%)	0.315
Age, years	84.0 (80.0, 86.0)	84.0 (81.0, 86.0)	0.611
BMI, kg/m^2^	23.7 (21.1, 25.9)	23.7 (20.4, 26.0)	0.242
ASA-PS			
3, n	91 (97.8%)	64 (94.1%)	
4, n	2 (2.2%)	4 (5.9%)	
NYHA ≥3, n	22 (23.7%)	11 (16.2%)	0.323
STS risk score, %	12.8 (9.6, 21.3)	13.2 (9.9, 21.8)	0.770
Medical history			
Hypertension, n	78 (83.9%)	54 (79.4%)	0.535
Diabetes mellitus, n	17 (18.3%)	18 (26.5%)	0.248
Af, n	28 (30.1%)	19 (27.9%)	0.861
Stroke, n	11 (11.8%)	15 (22.1%)	0.088
CAD, n	31 (33.3%)	20 (29.4%)	0.612
Antihypertensive agents			
ACE inhibitor, n	13 (14.0%)	11 (16.2%)	0.823
AT_1_ blocker, n	38 (40.9%)	26 (38.2%)	0.748
Alpha blocker, n	2 (2.2 %)	2 (2.9%)	1.000
Alpha-beta blocker, n	10 (10.8%)	8 (11.8%)	1.000
Beta blocker, n	14 (15.1%)	13 (19.1%)	0.527
Calcium antagonist, n	39 (41.9%)	34 (50.0%)	0.339
ARNI, n	4 (4.3%)	2 (2.9%)	1.000
Cardiac echocardiogram			
LVEF, %	65.0 (56.0, 69.0)	63.0 (55.5, 69.3)	0.792
mPG, mmHg	53.0 (39.0, 65.0)	51.0 (42.8, 63.5)	0.828
AVAi, cm^2^/m^2^	0.43 (0.36, 0.54)	0.46 (0.36, 0.51)	0.524
Vmax, m/s	4.8 (4.1, 5.2)	4.6 (4.2, 5.1)	0.616
Moderate or severe AR, n	63 (67.7%)	38 (55.9%)	0.140
Moderate or severe MR, n	52 (55.9%)	43 (63.2%)	0.418

Differences between the PIH and non-PIH groups were estimated using Fisher’s exact test for categorical variables and the Mann–Whitney test for continuous variables. Data are shown as number (percentage of each group) or median (25^th^–75^th^ percentile)

*BMI* body mass index, *ASA-PS* American Society of Anesthesiologists Physical Status, *NYHA*, New York Heart Association functional class, *STS* Society of Thoracic Surgeons, *Af* atrial fibrillation, *CAD* coronary artery disease, *ACE* angiotensin-converting enzyme, *AT*_*1*_ angiotensin II type 1 receptor, *ARNI* angiotensin receptor neprilysin inhibitor, *LVEF* left ventricle ejection fraction, mPG mean pressure gradient, *AVAi* aortic valve area index, *Vmax* maximal velocity, *AR* aortic valve regurgitation, *MR* mitral valve regurgitation

**Table 2:** Perioperative data of the patients

	**PIH group**	**Non-PIH group**	***P* value**
Dose of anesthetics for induction			
Rm, mg/kg	0.00 (0.00, 0.08)	0.03 (0.0, 0.08)	0.070
Prop, mg/kg	0.44 (0.00, 0.88)	0.00 (0.00, 0.73)	0.016*
Keta, mg/kg	0.45 (0.00, 0.74)	0.79 (0.37, 1.00)	<0.001*
Remi, µg/kg/min	0.20 (0.10, 0.20)	0.10 (0.00, 0.20)	0.002*
Fent, µg/kg	0.00 (0.00, 1.02)	1.07 (0.00, 1.74)	<0.001*
Type of valve			0.228
SAPIEN 3, n	73 (78.5%)	58 (85.3%)	
Evolut, n	20 (21.5%)	10 (14.7%)	
Duration of surgery, h	1.6 (1.4, 1.8)	1.6 (1.3, 1.8)	0.763
Duration of anesthesia, h	2.9 (2.5, 3.2)	2.8 (2.5, 3.1)	0.436
Blood loss, g/kg/h	0.2 (0.0, 0.3)	0.1 (0.0, 0.3)	0.754
Urine output, mL/kg/h	3.1 (1.8, 4.8)	3.9 (1.7, 4.8)	0.348
Fluid infusion, mL/kg/h	9.9 (7.9, 13.4)	11.0 (9.2, 13.5)	0.197
Length of ICU stay, day	2.0 (2.0, 2.0)	2.0 (2.0, 2.0)	0.632
hospital day, day	7.0 (6.0, 8.0)	6.0 (6.0, 7.0)	0.754
Hospital death, n	0 (0.0%)	1 (1.5%)	1.000
Perioperative stroke, n	1 (1.1%)	1 (1.5%)	1.000

Differences between the PIH and non-PIH groups were estimated using Fisher's exact test for categorical variables and the Mann–Whitney U-test for continuous variables. Data are presented as number (percentage of each group) or median (25^th^–75^th^ percentile)

*Rm* remimazolam, *Prop* propofol, *Keta* ketamine, *Remi* remifentanil, *Fent* fentanyl

^*^ significant difference

**Table 3:** Hemodynamic parameters from induction of general anesthesia until 20 min post-induction

	**PIH group**	**Non-PIH group**	***P* value**
Baseline			
SAP, mmHg	143.0 (127.0, 159.0)	158.5 (138.0, 173.3)	0.011*
MAP, mmHg	87.0 (78.3, 99.0)	95.0 (87.0, 102.8)	0.006*
DAP, mmHg	60.0 (52.0, 67.0)	63.5 (55.0,70.0)	0.042*
HR, bpm	71.0 (63.0, 78.0)	70.0 (64.8, 80.0)	0.587
Pre-tracheal intubation			
min SAP, mmHg	92.0 (72.0, 106.0)	115.0 (101.0, 128.8)	<0.001*
min MAP, mmHg	61.0 (500, 67.0)	71.0 (64.3, 79.3)	<0.001*
min DAP, mmHg	43.0 (37.0, 49.0)	51.0 (45.0, 55.0)	<0.001*
min HR, bpm	61.0 (53.0, 69.0)	63.0 (56.0, 70.5)	0.221
Post-tracheal intubation			
min SAP, mmHg	75.0 (66.0, 82.0)	104.0 (95.0, 116.3)	<0.001*
min MAP, mmHg	50.0 (43.3, 52.0)	67.0 (59.5, 75.0)	<0.001*
min DAP, mmHg	36(32.0, 40.0)	48.0 (41.0, 55.0)	0.001*
min HR, bpm	56.0 (49.0, 64.0)	61.0 (50.0, 70.0)	0.039*
Vasopressors use			
Ephedrine, mg	0.0 (0.0, 4.0)	0.0 (0.0, 0.0)	0.001*
Phenylephrine, mg	0.1 (0.0, 0.2)	0.0 (0.0, 0.05)	<0.001*
Noradrenaline, µg	0.0 (0.0, 0.0)	0.0 (0.0, 0.0)	0.194
Atropin, mg	0.0 (0.0, 0.0)	0.0 (0.0, 0.0)	0.293

Differences between the PIH and non-PIH groups were estimated using Fisher's exact test for categorical variables and the Mann–Whitney U-test for continuous variables. Data are presented as number (percentage of each group) or median (25^th^–75^th^ percentile)

*SAP* systolic arterial pressure, *MAP* mean arterial pressure, *DAP* diastolic arterial pressure, *HR* heart rate, *min* minimal

^*^ Sgnificant difference

The ROC curves revealed that the cut-off values for STS risk score and baseline MAP to predict the development of PIH were and 6.5% and 90 mmHg, respectively. The AUC values of the STS risk score and MAP were 0.515 and 0.626, respectively, indicating low accuracies.

The result of the multivariable logistic regression analysis to develop a predictive model for PIH in patients undergoing TAVI is shown in Table 4. Baseline MAP ≥90 mmHg (aOR: 0.413; 95% CI: 0.193–0.887; *p*=0.023) and higher doses of fentanyl (per 1-µg/kg increase, aOR: 0.619, 95% CI: 0.418–0.915; *p*=0.016) and ketamine (per 1-mg/kg increase, aOR: 0.163, 95% CI: 0.062–0.430; *p*=0.002) for anesthetic induction were significantly associated with a lower risk of the development of PIH. A higher dose of propofol (per 1-mg/kg increase, aOR: 3.240, 95% CI: 1.320–7.920; *p*=0.010) for anesthetic induction was significantly associated with a higher risk of the development of PIH. The area under the curve (AUC) for this model was 0.802.

**Table 4:** Predictive model for post-induction hypotension in patients undergoing transcatheter aortic valve implantation

	**aOR**	**95%CI**	***P* value**
(Intercept)	4.960	1.750, 14.00	0.003*
STS risk score ≥6.5%	3.030	0.908, 10.10	0.071
mPG ≥60 mmHg	1.610	0.724, 3.950	0.242
Baseline MAP ≥90 mmHg	0.413	0.193, 0.887	0.023*
Dose of rm for induction (per 1 mg/kg increase)	1.070	0.009, 126.0	0.977
Dose of prop for induction (per 1 mg/kg increase)	3.240	1.320, 7.920	0.010*
Dose of fent for induction (per 1 µg/kg increase)	0.619	0.418, 0.915	0.016*
Dose of remi for induction (per 1 µg/kg/min increase)	0.485	0.137, 1.710	0.261
Dose of keta for induction (per 1 mg/kg increase)	0.163	0.062, 0.430	0.002*

Multivariate logistic regression analyses were performed to develop predictive models for post-induction hypotension in patients who underwent transcatheter aortic valve implantation. No variance inflation factor value was up to 10, indicating no collinearity in the model. The area under the curve was 0.802 (95% CI: 0.732, 0.873)

*aOR* adjusted odds ratio, *CI* confidence interval, *STS* Society of Thoracic Surgeons, *mPG* mean pressure gradient, *MAP* mean arterial pressure, *Rm* remimazolam, *Prop* propofol, *Keta* ketamine, *Remi* remifentanil, *Fent* fentanyl

^*^ significant difference

**Fig 2:**
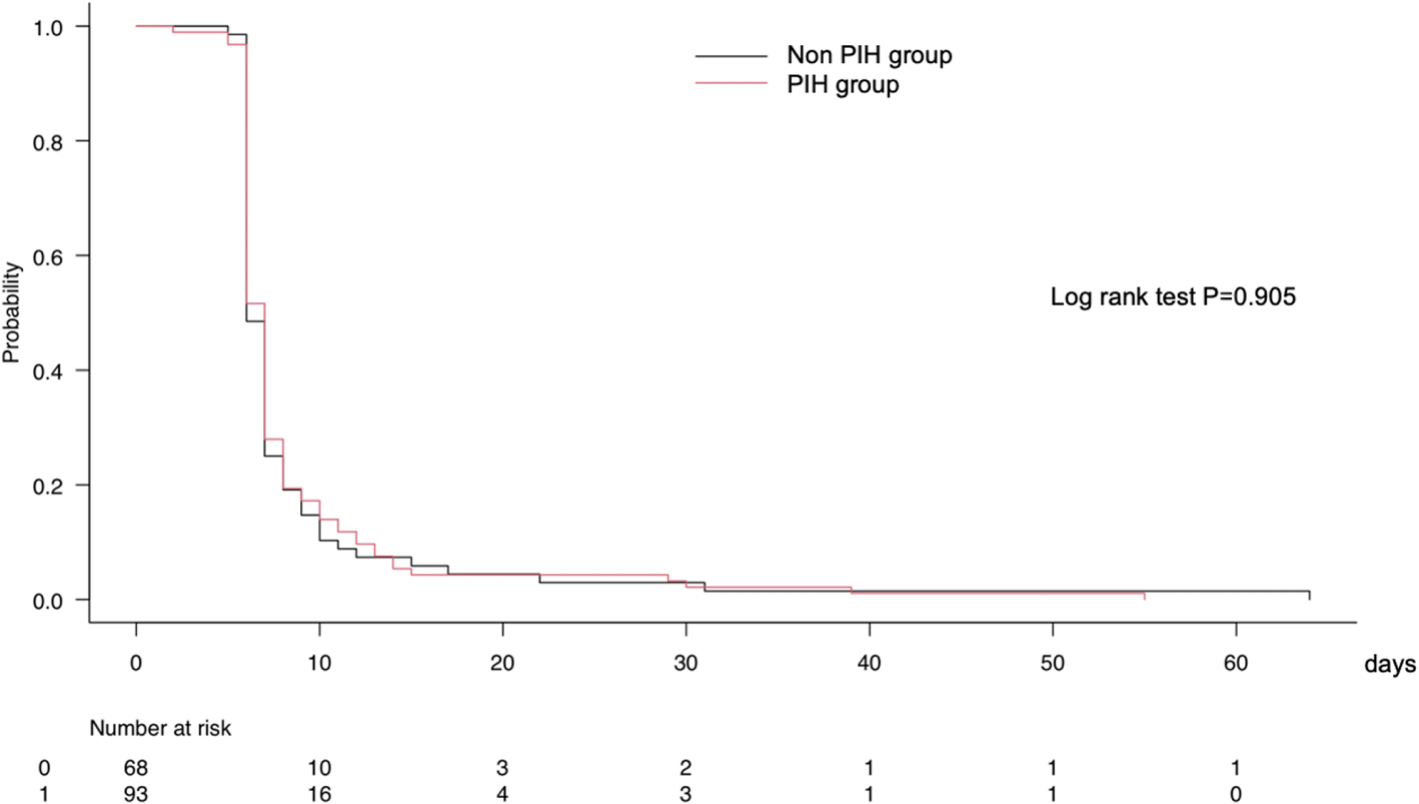
Kaplan–Meier curve analysis with log-rank test to compare the probability of hospital stay among the patients with and without PIH. PIH: post-induction hypotension; *, statistical significance

Figure 2 shows the results of the Kaplan–Meier curve analysis with log-rank test to compare the length of hospital stay between the PIH and non-PIH groups. There was no significant difference in the length of hospital stay between the two groups.

## Discussion

The present study developed a predictive model for PIH in TAVI patients. A baseline MAP ≥90 mmHg and higher doses of fentanyl and ketamine for anesthetic induction were significantly associated with a lower risk of developing PIH. A higher dose of propofol for anesthetic induction is significantly associated with a higher risk of developing PIH. The AUC value of this model was 0.802, indicating that its discrimination ability was good. There were no significant differences in the length of ICU and hospital stays between the PIH and non-PIH groups.

To the best of our knowledge, this is the first study to develop a predictive model for PIH in TAVI patients. In the present model, STS risk score was used as a variable for evaluating the preoperative condition. STS risk score was calculated using the patients' background such as age, sex, height, body weight, laboratory data, medical history, chronic medication, and cardiac function, and has been used to predict the mortality risk and complications after adult cardiac surgery [10]. Thus, we were able to adjust for many patient characteristics in the model, despite the small sample size. Additionally, the STS risk score is reported to be associated with mortality after TAVI [11]. However, the STS risk score was not a significant predictor of PIH in patients undergoing TAVI. Indeed, the short-term outcomes after TAVI were not significantly different between the PIH and non-PIH groups in the present study. However, as the 95% CI and p-value of STS risk score ≥6.5% were 0.908–10.10 and 0.071, respectively, increasing the sample size may change the results.

Consistent with the results of previous studies [4–6], baseline arterial pressure was also associated with PIH in this study. Although these studies included SAP as the baseline arterial pressure in the model, the present study included MAP as the baseline arterial pressure in the model. Generally, as patients who undergo TAVI are prone to have aortic regurgitation, the baseline arterial pressure should be assessed not only by SAP but also by DAP. Thus, MAP was included in the model and was an independent predictor of PIH in patients undergoing TAVI. In our institution, all antihypertensive agents were continued on the day of surgery because some of them are also used to treat heart failure. Therefore, caution is needed when interpreting this result.

In the present study, a higher dose of propofol for anesthetic induction was significantly associated with a higher risk of PIH. This result was consistent with that of previous studies [4, 12]. Propofol anesthesia is more likely to cause hypotension than remimazolam in ASA I or II patients [13]. Additionally, in the present study, higher doses of fentanyl and ketamine for anesthetic induction were significantly associated with a lower risk of PIH. A previous study showed that fentanyl with propofol induction was less likely to cause PIH than remifentanil with propofol induction [14]. However, an excessive amount of fentanyl for anesthetic induction can cause PIH. Indeed, a previous study showed that a higher dose of fentanyl can cause PIH [4]. The previous study showed that 1.51–5.0 µg/kg and >5.0 µg/kg of fentanyl for induction were more likely to cause PIH than 0–1.5 µg/kg of fentanyl for induction. In this study, the maximum dose of fentanyl was 4.27 µg/kg and the patient who was infused this dose of fentanyl was in the non-PIH group. The median doses (25^th^ to 75^th^ percentile) of fentanyl for induction were 0.00 (0.00, 1.02) µg/kg in the PIH group and 1.07 (0.00, 1.74) µg/kg in the non-PIH group, which means the patients in this study was treated with relatively low dose of fentanyl. Thus, clinicians need caution when interpreting this result about fentanyl. Ketamine is effective in maintaining hemodynamic stability during anesthetic induction [15]. However, ketamine can cause tachycardia and increase oxygen consumption owing to increased sympathetic stimulation [16, 17]. As these effects of ketamine are not preferable for patients with severe AS, it should be used carefully and with other anesthetics. Additionally, tracheal intubation under insufficient depth of anesthesia also causes sympathetic stimulation and adversely affects hemodynamics in patients with AS. Therefore, we do not recommend anesthetic induction with only ketamine and fentanyl in patients who undergo TAVI, because it tends to be insufficient in terms of the depth of anesthesia. Thus, it is important to use ketamine and fentanyl in addition to propofol or remimazolam for a well-balanced anesthetic induction.

This study has several limitations. First, as this was a single-center, retrospective observational study with a small sample size, selection bias and undetected confounding factors may have affected the results. Indeed, as the patients’ background was adjusted for only the STS risk score, we were not able to find any other variables that were associated with PIH except for baseline MAP. Second, as the choice of anesthetics was not standardized and was up to the anesthesiologist’s discretion, the development of PIH may have depended on the competence of the anesthesiologist. However, all cases were managed or supervised by anesthesia specialists. Third, as we did not follow the patients’ courses after hospital discharge, postoperative complications after hospital discharge and long-term outcomes could not be assessed. Although the prevalence of postoperative complications was not significantly different between the PIH and non-PIH groups, PIH might affect the long-term outcomes.

In conclusion, this study developed a predictive model for PIH in patients undergoing TAVI. A baseline MAP ≥90 mmHg and higher doses of fentanyl and ketamine for anesthetic induction were significantly associated with a lower risk of PIH. Of course, excessive amount of these two drugs should be avoided. A higher dose of propofol for anesthetic induction is significantly associated with a higher risk of PIH. This model may be helpful for anesthesiologists in preventing PIH in patients undergoing TAVI. However, as this was a single-center, retrospective observational study with a small sample size, these results should be treated with caution and further studies are required to strengthen this evidence.

## Data Availability

Data will be made available on reasonable request.

## Abbreviations

AS: Aortic stenosis
SAVR: Surgical aortic valve replacement
TAVI: Transcatheter aortic valve implantation
PIH: Post-induction hypotension
ASA-PS: American Society of Anesthesiologists physical status
MAC: Monitored anesthesia care
SAP: Systolic arterial pressure
NYHA: New York Heart Association
STS: Society of Thoracic Surgeons
HR: Heart rate
ICU: Intensive care unit
ROC: Receiver operating characteristic
VIF: Variance inflation factor
AUC: Area under the curve
OR: Odds ratios
CI: Confidence interval
MAP: Mean arterial pressure
DAP: Diastolic arterial pressure
